# Lipid Metabolism Alterations in a Rat Model of Chronic and Intergenerational Exposure to Arsenic

**DOI:** 10.1155/2019/4978018

**Published:** 2019-10-15

**Authors:** Cesar Rivas-Santiago, Irma González-Curiel, Sergio Zarazua, Michael Murgu, Alonso Ruiz Cardona, Blanca Lazalde, Edgar E. Lara-Ramírez, Edgar Vázquez, Julio Enrique Castañeda-Delgado, Bruno Rivas-Santiago, Jesús Adrián Lopez, Alberto R. Cervantes-Villagrana, Yamilé López-Hernández

**Affiliations:** ^1^CONACyT, Unidad Académica de Ciencias Biológicas, Universidad Autónoma de Zacatecas, Zacatecas, Mexico; ^2^Maestría en Ciencia y Tecnología Química, Unidad Académica de Ciencias Químicas, Universidad Autónoma de Zacatecas, Zacatecas, Mexico; ^3^Laboratorio de Neurotoxicología, Facultad de Ciencias Químicas, Universidad Autónoma de San Luis Potosí, San Luis Potosí, Mexico; ^4^Waters Technologies of Brazil, Barueri, Brazil; ^5^Unidad de Investigación Biomédica de Zacatecas, Instituto Mexicano de Seguro Social, Zacatecas, Mexico; ^6^Waters Corporation, Mexico City, Mexico; ^7^Catedrático-CONACYT, Unidad de Investigación Biomédica de Zacatecas-IMSS. Zacatecas, Zacatecas, Mexico; ^8^Laboratorio de microRNAs, Unidad Académica de Ciencias Biológicas, Universidad Autónoma de Zacatecas, Zacatecas, Mexico

## Abstract

Chronic exposure to arsenic (As), whether directly through the consumption of contaminated drinking water or indirectly through the daily intake of As-contaminated food, is a health threat for more than 150 million people worldwide. Epidemiological studies found an association between chronic consumption of As and several pathologies, the most common being cancer-related disorders. However, As consumption has also been associated with metabolic disorders that could lead to diverse pathologies, such as type 2 diabetes mellitus, nonalcoholic fatty liver disease, and obesity. Here, we used ultra-performance liquid chromatography (UPLC) coupled to electrospray ionization/quadrupole time-of-flight mass spectrometry (ESI-QToF) to assess the effect of chronic intergenerational As exposure on the lipid metabolism profiles of serum from 4-month-old Wistar rats exposed to As prenatally and also during early life in drinking water (3 ppm). Significant differences in the levels of certain identified lysophospholipids, phosphatidylcholines, and triglycerides were found between the exposed rats and the control groups, as well as between the sexes. Significantly increased lipid oxidation determined by the malondialdehyde (MDA) method was found in exposed rats compared with controls. Chronic intergenerational As exposure alters the rat lipidome, increases lipid oxidation, and dysregulates metabolic pathways, the factors associated with the chronic inflammation present in different diseases associated with chronic exposure to As (i.e., keratosis, Bowen's disease, and kidney, liver, bladder, and lung cancer).

## 1. Introduction

Arsenic (As) is a chemical metalloid widely distributed in nature from soil to groundwater; in its inorganic (iAs) form, it can be highly toxic, especially trivalent arsenic (iAsIII) and oxidized arsenate (iAsV). Around the world, people are constantly exposed to this chemical through the consumption of contaminated water and the use of contaminated water in agriculture, which contaminates food. For example, the concentration of As detected in drinking water in countries in South Asia, as well as countries such as Argentina, Australia, Hungary, Mexico, and the United States, is higher than the 10 *μ*g/L permissible limit established by the World Health Organization (WHO) [1–3].

Chronic consumption of As has been associated with diverse diseases, including skin, lung, bladder, and liver cancers [1], cognitive deficiencies [4], cardiovascular diseases [5], hypertension [6], diabetes mellitus [7–9], and anemia [10]. Epidemiological studies identify As as a risk factor for human health [11–13]; until now and even with the vast number of studies performed (reviewed in [14]), it is not well known the molecular mechanisms of the toxic and carcinogenic effects of As. In addition, the number of metabolic pathways altered by As is too extensive to fully understand, and there is not a suitable animal model for the study of the long-term toxicity effects of As exposure [15].

Some of the biochemical pathways altered by As exposure are glucose metabolism [16, 17], protein metabolism [18], choline metabolism [19], and membrane phospholipid degradation, which has direct implications in apoptosis [20]. These metabolic abnormalities could be the result of an endocrine signalling disruption. For example, recent studies *in vivo* have demonstrated the adverse effects of As exposure on steroid receptors and endogenous/exogenous hormone-driven genes [21]. Environmental pollutants, such as As, play a role in the generation of oxidative stress in cells [22–24]. The assessment of lipid oxidation is an important marker of oxidative damage that is associated with membrane phospholipid breakdown, indicative of cellular damage. Concentrations of malondialdehyde (MDA) reflect the intensity of lipid oxidation and have been used by different authors as a biochemical biomarker of oxidative stress [25, 26].

Chronic exposure to arsenic has been associated with alterations in the proteome [27, 28] and metabolome in humans [29–32] and in murine models [33–41]. The mechanism underlying these alterations is not well understood but could be associated with disruption of some biological pathways through epigenetic modifications [42–46]. Metabolomic studies are becoming popular to explore the altered pathway consequences of long-term exposures that ultimately lead to the development of various diseases. Previous metabolomic studies reported the adverse effects of acute exposure to high As levels (7 days of exposure to 3 mg/kg/day) [33], of chronic exposure to high As levels (50 ppm for 6 months) [40], and of chronic exposure to lower As (0.5, 2, and 10 ppm for 3 months) [41] in murine models. Overall, these studies have provided valuable information on the toxicity and the biochemical pathways altered by As and have reflected real-life exposure to As as it occurs in human populations.

The study of lipid metabolism is crucial in chronic diseases and metabolic syndrome, where an inflammatory process takes place. Adiponectin is an adipocyte-derived hormone that increases insulin sensitivity and has an important role in the regulation of lipid metabolism, in an insulin-independent manner [47]. Besides its role in lipid metabolism, this hormone has beneficial properties, such as anti-inflammatory and antioxidant. However, the utility of adiponectin as a candidate biomarker in metabolic syndrome prediction and risk assessment seems paradoxical across studies with uncertain hazard extents for potential differences regarding ethnic origin, gender, samples, comparisons, obesity, or other disease status [48].

In this study, we used ultra-performance liquid chromatography (UPLC)-mass spectrometry to analyse the lipid metabolism of rats exposed to 3 ppm of As in drinking water (approx. 0.4 mg/kg) from gestation, lactation, and throughout the development until adulthood. The As concentration used in this work has been used in previous studies [4, 49, 50] and did not show toxic effects on body weight, diminution of brood size, or mortality. This model tries to simulate a real-life exposure condition, where people are chronically exposed to As throughout their lifetimes; since the placenta is not a barrier to As [19], intergenerational effects of As exposure on lipid metabolism could be induced.

## 2. Clinical Significance

The present study

- Establishes the usefulness of untargeted lipidomics to identify possible biomarkers of exposure to As
- Establishes that increased levels of the lysophosphatidylcholines LysoPC (20:4), LysoPC (15:0), LysoPC (16:0), and LysoPC (18:1) are associated with As exposure
- Establishes that chronic exposure to As increases lipid oxidation, which could be associated with inflammatory processes that lead to chronic diseases, such as cancer, atherosclerosis, and diabetes
- Indicates an effect of chronic and intergenerational As exposure on lipid alterations, which may be evaluated and extrapolated in cross-sectional studies

## 3. Materials and Methods

### 3.1. Reagents

High-performance liquid chromatography- (HPLC-) grade isopropanol, acetonitrile, methanol, and water were purchased from JT Baker (Brick Town, NJ, USA). High-purity formic acid (99%) and ammonium acetate (99%) were provided by Thermo Scientific (Rockford, IL, USA), and sodium arsenite was obtained from Sigma-Aldrich (St. Louis, MO, USA).

#### 3.1.1. The Animal Model for Chronic as Exposure

All procedures performed in studies involving animals were done in accordance with the ethical standards of the Autonomous University of San Luis Potosi and were conducted according to the Guide for the Care and Use of Laboratory Animals. This research was conducted under the approval of Ethics Committee in Research and Teaching from Chemistry Faculty of Autonomous University of San Luis Potosi, Mexico (No. CEID2013-010).

Eight female and four male adult Wistar rats weighing between 250 and 300 g were randomly placed in four cages in a ratio of two females to one male and maintained in the animal facilities under controlled conditions of light (12 hours light/dark cycle), temperature, and humidity. Commercial food (LabDiet 5001, St. Louis, MO, USA) and water were provided *ad libitum*. As exposure conditions were carried out according to previous reports [51]. In brief, a group of three (two females and one male) animals received drinking water containing 3 ppm of arsenic as sodium arsenite (Sigma-Aldrich, St. Louis, MO, USA), approximately 0.3 to 0.4 mg arsenic/kg/day and were identified as the experimental group. The control group received pure drinking water. Rats from the experimental and control groups were weighed daily, until weight changes indicated pregnancy status. After pregnancy detection, rats were transferred to separate cages, where arsenic exposure continued throughout gestation and lactation. The broods were weaned after one month and separated by sex. Offspring from experimental (five female rats: AsF; eight male rats: AsM) and control groups (five female rats: CF; eight male rats: CM) continued their treatments until the age of 4 months. At this age, rats from both groups were euthanized by decapitation. Blood was collected, and serum was isolated by centrifugation and maintained at −70°C until its use.

#### 3.1.2. Body Weight and Total Serum Adiponectin Determination

Rats were weighed at the experimental endpoint with a technical balance. Weights were recorded in grams (g). Total serum adiponectin was assessed by enzyme-linked immunosorbent assay (ELISA) in 96-well plates (Abcam®, Cambridge, MA, USA) according to the manufacturer's instructions. In brief, standards or samples were added (50 *μ*L/well) to precoated and blocked 96-well plates. The plates were incubated for 1.5 h and washed 5 times with washing buffer (200 *μ*L/well). Biotinylated detection antibody was added, and the plates were incubated for 1.5 h and washed again. Streptavidin-peroxidase conjugate was added (50 *μ*L/well), and the plates were incubated for 30 min at room temperature. After a final washing step, the plates were incubated with chromogen substrate (50 *μ*L/well) for 15 min. Then, stop solution was added (50 *μ*L/well), and wells were read in triplicate using a Fisher Scientific Multiskan FC microplate reader (450 nm, with a correction wavelength of 650 nm).

#### 3.1.3. Sample Preparation for Mass Spectrometry

Serum lipid extraction was carried out as described previously [52]. In brief, serum samples were thawed on ice. Immediately, 100 *μ*L of serum was extracted with 300 *μ*L of precooled isopropanol, vortexed for 1 min, and incubated at room temperature for 10 min. The extraction mixture was then stored at −20°C overnight. After centrifugation at 10 000 g for 20 min, the supernatants were collected, and aliquots of 25 *μ*L were prepared and stored at −80°C. One aliquot was transferred into LC vials and diluted to 1 : 20 with isopropanol/acetonitrile/water (2 : 1 : 1, v : v : v). Additionally, pooled serum samples were prepared by combining 10 *μ*L of individual sera and were identified as quality controls (QC). These pooled samples were subjected to the same procedure as above.

#### 3.1.4. Ultra-Performance Liquid Chromatography (UPLC)-Mass Spectrometry Method for Lipidomic Analysis

Samples were analysed as described previously [53, 54]. In brief, ACQUITY UPLC I-Class (Waters Corp., Milford, MA, USA) coupled to a XEVO-G2 XS quadrupole time-of-flight (ToF) mass spectrometer (Waters, Manchester, NH, USA) with an electrospray ionization source was used. The separation of different lipid classes was done using an UPLC BEH C18 column (2.1 × 100 mm, 1.7 *μ*m) using binary gradient elution of solvents A and B. The mobile phase was A: 10 mM ammonium acetate with 0.1% formic acid in acetonitrile/water (60 : 40, v : v); and B: 10 mM ammonium acetate with 0.1% formic acid in isopropanol/acetonitrile (90 : 10, v : v). The mobile phase was delivered at a flow rate of 0.4 mL/min, initially with 60% A, followed by a linear gradient to 57% A over 2 minutes, and then the percentage of A was decreased to 50% within 0.1 min. Over the next 9.9 minutes, the gradient was ramped to 46% A, and the amount of A was then decreased to 30% in 0.1 min. Over 5.9 minutes, the amount of A decreased to 1% and returned to initial conditions (60%) at the end of 20 minutes. The column temperature was adjusted to 55°C. The injection volume was 5 *μ*L.

Data were acquired in positive electrospray ionization (ESI+) mode with the capillary voltage set to 2.0 kV, the cone voltage to 30 eV, and the source temperature to 120°C. The desolvation gas was nitrogen, with a flow rate of 800 L·h^−1^ and temperature of 550°C. Data were acquired from *m/z* 100 to 2000 in MS^E^ mode in which the collision energy was alternated between low energy (5 eV) and high energy (ramped from 15 to 30 eV). As a lock mass for accurate mass measurements, leucine enkephalin (200 pg/*μ*L in acetonitrile : water (50 : 50 v/v) + 0.1% formic acid) was infused. For calibration, 0.5 mM sodium formate was used. Four pooled samples (QC) were initially injected to equilibrate the column. One QC sample was injected every five samples.

#### 3.1.5. Data Acquisition and Statistical Analysis

The raw MS^E^ datasets were acquired in continuum mode and processed within UNIFI 1.8.1 (Waters Corp., Milford, USA). The analysis parameters were as follows: retention time of 0.5–18 min and peak width of 1–30 s. Data within UNIFI 1.8.1 were passed through the apex peak detection and alignment processing algorithms. The intensity of each ion was normalized with respect to the total ion count (TIC) to generate a data matrix that consisted of the retention time, *m/z* value, and the normalized peak area. In the univariate analysis, the D'Agostino-Pearson test was performed to know whether the data showed a normal distribution. A *t* test with Welch's correction was performed to compare exposed and nonexposed animals. This test was used to account for the unequal variations between the two groups, as identified using Levene's test. Values with *p* < 0.05 were considered statistically significant. The analysis was performed with SPSS v22 software (Chicago, IL, USA).

The multivariate data matrix was analysed by using EZinfo software (Waters Corp., Milford, MA, USA) and MetaboAnalyst [55]. The data were mean-centred and Pareto-scaled prior to principal component analysis (PCA) and orthogonal projection to latent structures discriminant analysis (OPLS-DA). Potential markers of interest were extracted from the combining variable importance projection (VIP) plot that was constructed from the loading plots of OPLS-DA.

The XCMS Online platform (La Jolla, CA) was also used for metabolic pathway screening (https://xcmsonline.scripps.edu). The LC-MS/MS raw data from the Waters format were transformed to the mzXML format with ProteoWizard software. Then, the mzXML files were uploaded into the XCMS Online tool. Using the XCMS Online graphical interface, the pairwise comparison parameters for female rats (AsF vs CF) and male rats (AsM vs CM), as well as a comparison between AsF and AsM, were selected to evaluate differential pathways and the related metabolites that could influence differential response after arsenic exposure.

Differences between experimental groups were evaluated by the unpaired *t*-test with unequal variance with post hoc Benjamini-Hochberg (FDR) and Bonferroni corrections. The level of statistical significance was set at 95% (*p* < 0.05).

#### 3.1.6. Compound Identification

The high-resolution LC-MS/MS features that were found to be significant in class separation were further identified by searching accurate masses against the online available databases, LIPID MAPS (http://www.lipidmaps.org/), METLIN (https://metlin.scripps.edu), and the Human Metabolome Database (http://www.hmdb.ca). The identity of compounds was confirmed by the study of accurate mass and isotopic distributions for the precursor and product ions formed by collision induced fragmentation (CID) as previously described; a 10 ppm mass tolerance for the precursors and for theoretical fragmentation searching was established.

Due to the high number of lipid isomers, identification based only on accurate mass is not enough for an unambiguous identification; for many lipids, the database search returned several possible compounds. To define the best hits, other experimental data were used to remove incorrect candidate structures. These data included retention time, since the chromatographic method used for the analysis has a well-established retention time range for each lipid class, and the fragments generated by the high-collision energy scans on MS^E^ analysis, compared with *in silico* fragmentation generated by Unifi software and by manual assignment of fragments based on known fragmentation patterns. However, the accurate precursor and fragment mass data are not enough for the complete assignment of a lipid structure, since double-bond position and configuration on fatty acid chains, as well the position of fatty acid chains for lipids containing more than one chain (e.g., PC and TG), could only be confirmed by injecting reference standards that were not available for this work. Fragmentation data provided a good confirmation of the number of carbon atoms and number of double bonds for the fatty acid chain; some examples for the main lipid classes found in the samples are shown in the supplementary information.

#### 3.1.7. Determination of Lipid Oxidation

Malondialdehyde (MDA) concentrations were measured as thiobarbituric acid reactive substances (TBARS) according to a modified version of the procedure previously described [56]. In brief, 250 *μ*L of serum was mixed with 1 mL of 1/12 NH_2_SO_4_ and gently shaken. Then, 0.3 mL of 10% phosphotungstic acid and 1 mL of thiobarbituric acid (0.6%) were added to the tube and heated in a boiling water bath for 1 h. The samples were cooled at room temperature. The resulting chromogen was extracted with 1.5 mL *n*-butyl alcohol by vigorous shaking. The organic phase was separated by centrifugation at 1600 g for 10 min, and its absorbance was recorded at a wavelength of 530 nm. The level of absorbance was converted into nmol/mL MDA from a standard curve generated with 1,1,3,3-tetraethoxypropane (Sigma-Aldrich, St. Louis, MO).

## 4. Results

### 4.1. Effects of as Exposure on Body Weight and Serum Adiponectin

**Figure 1:**
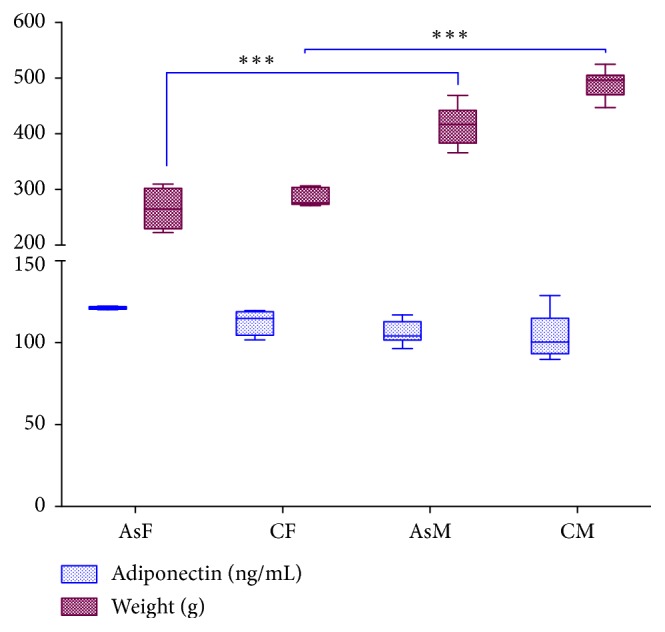
Effects of As exposure on body weight and serum adiponectin. Female rats (AsF and CF) had nonsignificant higher levels of adiponectin and lower body weight than male rats (AsM and CM). Significant differences (*p* = 0.001) were found between male and female rats either in exposed or nonexposed groups (two-way ANOVA with Tukey's multiple comparison test).

In the current study, no differences were found in water or food consumption, no changes in body weight were observed in the offspring born from female rats exposed to As, and no morphological changes were observed during the development of the pups. Adult exposed rats had lower body mass than nonexposed rats, although the differences were nonsignificant (*p* > 0.05). However, body weights of female rats were significantly lower (*p* < 0.05) than male rats either in exposed or nonexposed groups. Also, nonsignificant changes (*p* > 0.05) were observed in the total level of serum adiponectin in exposed and nonexposed rats. However, a trend towards an increase was observed, as was higher adiponectin level, in female rats compared to male rats (Figure 1). Interestingly, the level of serum adiponectin correlated negatively with body weight. Thus, animals with higher serum adiponectin had lower body weight (Figure 1).

### 4.2. Lipidomic Results

The chromatographic method used for lipid separation has been used before by our group [54] and showed good retention time reproducibility and was able to perform the separation between lipid classes, as well as within classes, with good separation of positional isomers (Supplementary [Supplementary-material supplementary-material-1]). The method's reproducibility was evaluated between quality control (QC) injections, which were run before the samples, and showed excellent reproducibility for retention time, peak shape, and peak intensity. These were evaluated by direct comparison of overlaid chromatograms (Supplementary Figure 2) that showed no drifts in retention time or intensity, reflecting the stability and reproducibility of the system.

**Figure 2:**
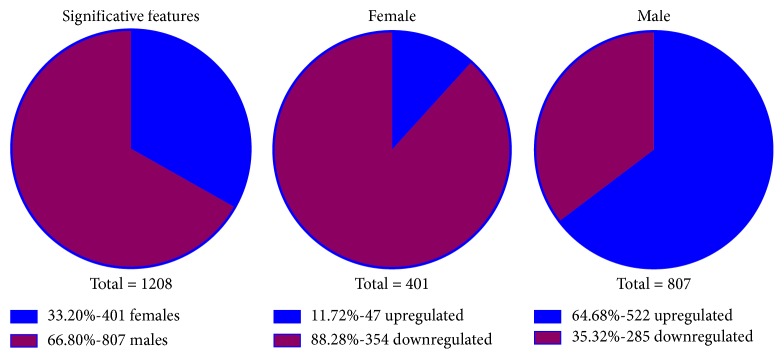
Metabolic features altered by chronic arsenic exposure. The number of significant alterations is approximately 2-fold in male than in female rats exposed to As. More metabolites are upregulated in male rats compared to female rats, while more metabolites are downregulated in female than in male exposed rats. *T*-test with Welch's correction was performed to account for the unequal variations between the two groups.

After the analysis with XCMS online, with criteria of *p* < 0.05 and VIP >1.5, we found that 401 and 807 metabolic features were significantly altered in AsF and AsM, respectively. Among the 401 metabolic features significantly changed (*p* < 0.05) in the group of female rats treated with arsenic, 354 were downregulated and 47 upregulated. Similarly, from the 807 metabolic features altered in the group of male rats treated with arsenic, 522 were upregulated and 285 downregulated (Figure 2). The results are available in the XCMS online platform (Job IDs: 1200223, 1200505, and 1200737).

In a parallel analysis, a total of 1155 features were detected under the conditions employed for the preprocessing of the raw data within UNIFI 1.8.1 (Waters Corp., Milford, MA, USA) and detailed in the Materials and Methods section. In the PCA score plot, the samples of control groups were only partially separated from treated groups and separated regarding sex differences. PCA was first performed to discover intrinsic treatment-related clusters within the datasets. Heat map was also built with the data acquired in positive mode, reflecting differences regarding sex and in some metabolites when compared between treated and unexposed groups (Supplementary Figure 3).

Following this, partial least-squares discriminant analysis (PLS-DA) and orthogonal partial least-squares discriminant analysis (OPLS-DA) were used to improve separation among the groups and screen biomarkers.

**Figure 3:**
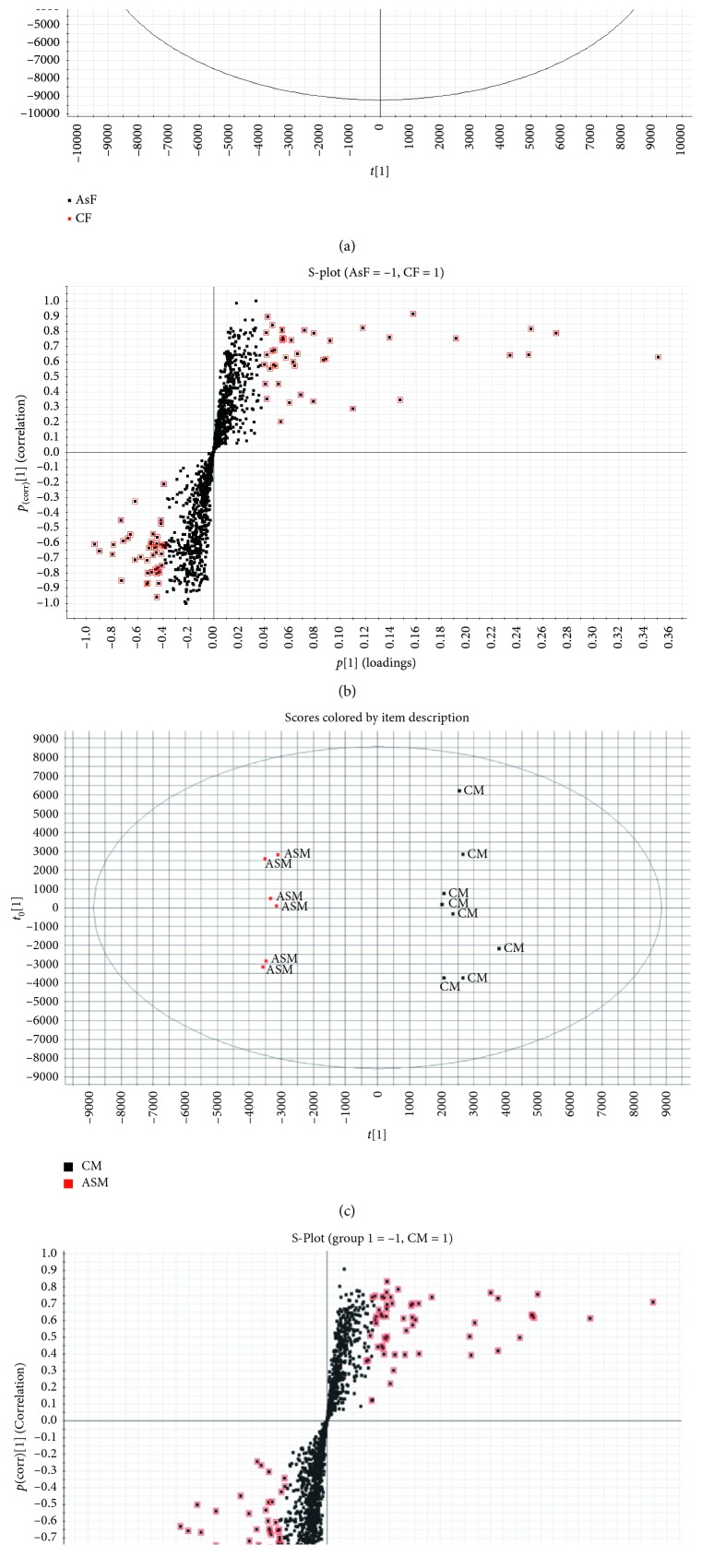
Orthogonal partial least-squares discriminant analysis (OPLS-DA) of determinants in positive and negative ionization modes. (a) Score plot from OPLS-DA shows group separation between control male rats (CM) and As-exposed male rats (AsM), with quality of variance explained and variance predicted (*R*^2^ (cum) = 99%, *Q*^2^ (cum) = 89%). (b) OPLS-DA loadings S-Plot comparing features from CM rats and AsM male rats in positive ionization mode. (c) Score plot from OPLS-DA shows group separation between control female rats (CF) and As-exposed female rats (AsF), with quality of variance explained and variance predicted (*R*^2^ (cum) = 99%, *Q*^2^(cum) = 85%). (d) OPLS-DA loadings S-Plot comparing features from CF rats and AsF rats in positive ionization mode. Each spot in S-Plot corresponds to a feature with characteristic *m/z* ratio and retention time. The metabolites marked in red were treated as putative biomarkers of As exposure.

The OPLS-DA score plots resulted in unambiguous intergroup separation. The parameters of the obtained models were satisfied with good quality of variance explained (*R*^2^) and variance predicted (*Q*^2^) and are represented in Figure 3. The loading plots (S-Plot) identified the metabolites with significant differences in abundance between the study groups. Differential metabolites were selected based on the separation through the OPLS-DA loadings and VIP. VIP represents the extracted variables' ability to discriminate between different treatments, and the variables with VIP values greater than 1.5 were included in the set of biomarkers analysed.

Lipids that were selected in the OPLS-DA loading S-Plot were identified as described in Materials and Methods. Identified lipids with significant changes in expression in female and male rats exposed to arsenic are summarized in Table 1. The exact measured mass, mass error (ppm), retention time, detected lipid, MS^E^ fragments, and percentage of changes between groups (fold change), along with the statistical significance of each change, are detailed in the table. Metabolomic profiling of serum samples showed statistically significant changes in glycerophospholipid and glycerolipid metabolism (*p* < 0.05). A decrease in the levels of phosphatidylcholines (PCs) and an increase in the levels of lysophosphatidylcholines (LysoPCs) were found in the animals exposed to As and were found to be a more evident effect in male rats.

The selected lipids were identified and classified according to their degree of physicochemical and/or spectral similarity to published data. MS^E^ data were manually inspected for the correct identification of major ions, in conjunction with the use of Lipid MS Predictor software (http://www.lipidmaps.org/).

**Figure 4:**
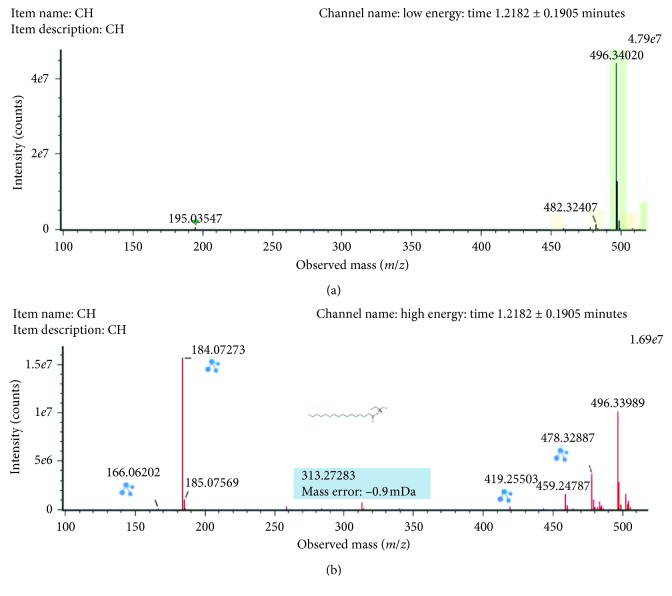
Low-collision (a) and high-collision (b) energy exact mass spectra of LysoPC (16:0). The low-energy spectrum only contains the precursor ion at *m/z* 496.34029, whereas in the high-energy spectrum, various diagnostic fragments appear as the loss of the FA chain at 258.1101. Also, the major fragment ion at *m/z* 184.07273 can be seen corresponding to the polar head group.

The *m/z* 496.3396 (ESI+, RT = 1.21 min) with a reported prominent fragment ion at *m/z* 184.0725 was annotated as LysoPC (16:0) (Figure 4). The other minor fragments (*m/z* 258.1088, *m/z* 313.2854, *m/z* 419.3284, and *m/z* 478.3284) were also in agreement with experimental and *in silico* MS/MS data described in the database METLIN for this compound. LysoPC (16:0) was highly upregulated in male rats exposed to As in comparison with control rats.

**Figure 5:**
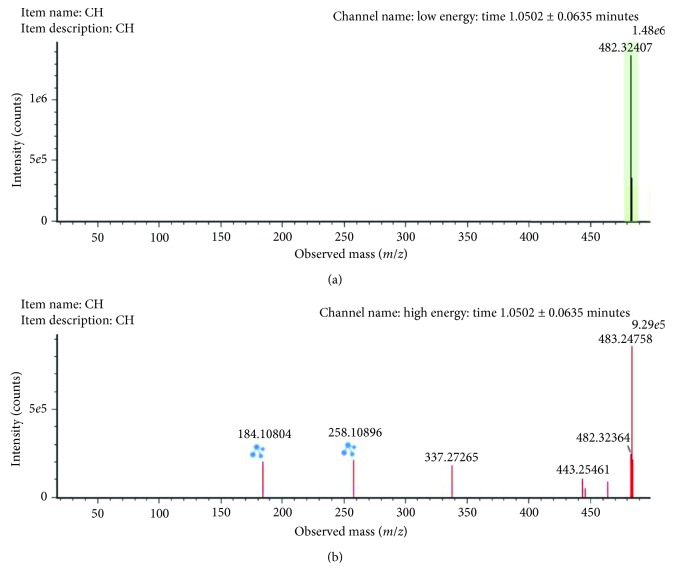
Low-collision (a) and high-collision (b) energy exact mass spectra of LysoPC (15:0). The low-energy spectrum only contains the precursor ion at *m/z* 482.32407, whereas in the high-energy spectrum, various diagnostic fragments appear as the loss of the FA chain at 258.10896. Also, the major fragment ion at *m/z* 184.10804 can be seen corresponding to the polar head group.

The *m/z* 482.32407 (ESI+, RT = 1.05 min) with a reported prominent fragment ion at *m/z* 184.0725 was annotated as LysoPC (15:0) (Figure 5). The other minor fragment (*m/z* 258.10896) corresponded to the acyl loss. LysoPC (15:0) was highly upregulated in male rats exposed to arsenic in comparison with control rats.

**Figure 6:**
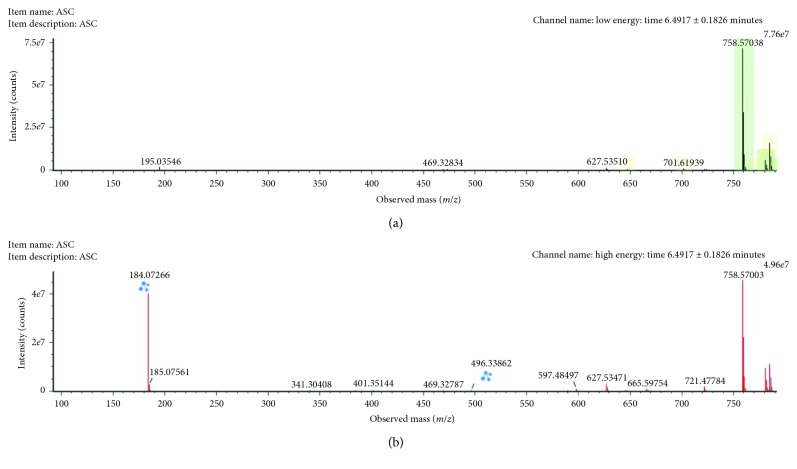
Low-collision (a) and high-collision (b) energy exact mass spectra of PC (16:0/18:2). The low-energy spectrum only contains the precursor ion at *m/z* 758.57038, whereas in the high-energy spectrum, various diagnostic fragments appear as the loss of the various FA chains at *m/z* 496.3410 and 478.32789 (the water loss) and 502.32807, which belong to the water loss of the chain at *m/z* 520.3407. Also, the major fragment ion at *m/z* 184.0733 can be seen corresponding to the polar head group.

The *m/z* 758.5711 (ESI+, RT = 6.49 min) with a reported prominent ion at *m/z* 184.0725 was annotated as PC (16:0/18:2) (Figure 6). The other minor fragment (*m/z* 496.33862) corresponded to the acyl loss. PC (16:0/18:1) was downregulated in male rats exposed to arsenic in comparison with control rats.

**Figure 7:**
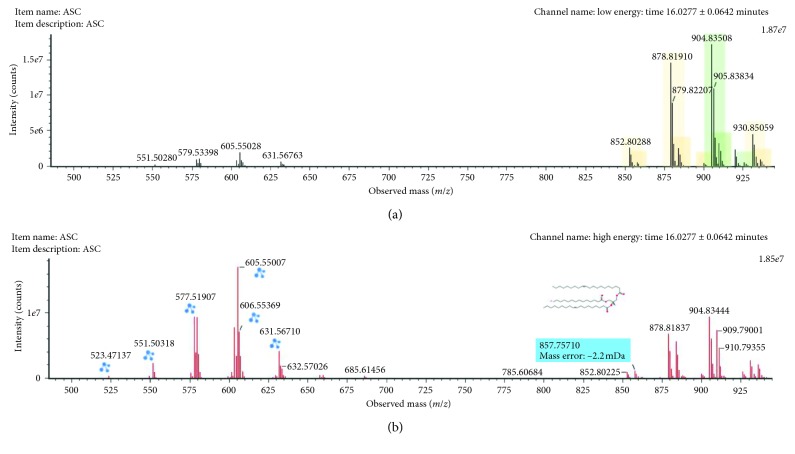
Low-collision (a) and high-collision (b) energy exact mass spectra of TG (16:0/18:1/20:1). The low-energy spectrum only contains the precursor ion at *m/z* 904.53508, whereas in the high-energy spectrum, various diagnostic fragments appear as the loss of the various FA chains at 631.56710, 605.55007, 577.51907, and 520.3407.

The *m/z* 904.8347 (ESI+, RT = 16.03 min) was annotated as TG (16:0/18:1/20:1) (Figure 7). Three fragments were generated as a consequence of the rupture of acyl chains (*m/z* 631.56710, 605.55007, and 577.51907). TG (16:0/18:1/20:1) was downregulated in male rats exposed to arsenic in comparison with control rats.

From the global metabolomic analysis conducted in XCMS software, it was predicted that two metabolic pathways were dysregulated, namely, the thyroid hormone metabolism II (via conjugation and/or degradation) and the ubiquinol-10 biosynthesis pathway.

### 4.3. Determination of Lipid Oxidation

**Figure 8:**
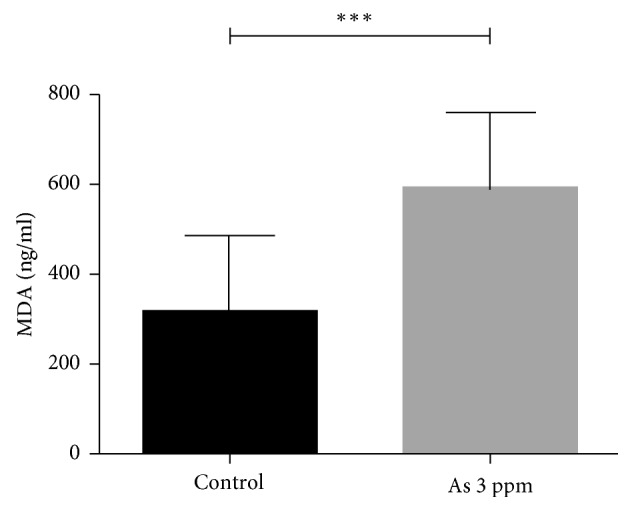
Lipid oxidation (MDA level) in the serum of rats exposed to 3 ppm of As in drinking water. ^*∗∗∗*^*p* = 0.0002 vs. control (*T*-test).

To evaluate the effects of As exposure on lipid oxidation, we carried out an analysis of lipid oxidation in serum. Lipid oxidation was increased in treated animals with respect to the controls (*p* < 0.05), as determined by the MDA method.  Figure 8 shows the results of lipid oxidation determination.

## 5. Discussion

In the present study, we found that chronic and intergenerational exposure to As has an effect on the lipid metabolism, significantly increasing the levels of the lysophosphatidylcholines: LysoPC (20:4), LysoPC (15:0), LysoPC (16:0), and LysoPC (18:1). Chronic exposure to As also increases lipid oxidation, which could be associated with inflammatory processes that lead to chronic diseases. Previously, Wang et al. provided novel evidences to support the association between As exposure and metabolic disruption, contributing to understanding the mechanism of chronic arsenic toxicity. Possible role of lipid alterations and their precursors, mediating inflammatory process associated with toxicity after As exposure, is briefly speculated in the present work.

Here, the effect of chronic and intergenerational As exposure on rat lipid metabolism was evaluated in an *in vivo* model. The model of exposure employed in the present work has been well characterized by our group [51, 57–60] and represents an important reference for neurotoxicity and oxidative damage. In these previous articles, all the As quantifications were performed in brain tissue of As-exposed rats through validated methods in our laboratory such as atomic fluorescence or by inductively coupled plasma-sector field mass spectrometry (ICP-SFMS), and NIST standards were used to assess quality assessments and quality controls.

To our knowledge, although several studies have focussed on the reproductive and carcinogenic effects of transplacental As exposure [61–63], scarce data are available about the intergenerational effects of arsenic on lipid metabolism in animal models. States and colleagues demonstrated that transplacental arsenic exposure in mice alters developmental programming in the foetal liver, leading to an enduring stress and proinflammatory response postnatally, which may contribute to early onset of atherosclerosis [64]. Ditzel and colleagues demonstrated that *in utero* and continuous early-life exposure to AsIII disrupted normal metabolism and elevated the risk for fatty liver disease in mice maintained on a high-fat diet, suggesting that individuals exposed to AsIII during key developmental periods and who remain exposed through eating a Western-style diet may be at increased risk for metabolic disease later in life [65].

Arsenic influences many diverse disease processes, such as cell signalling, cell cycle control, oxidative stress, and DNA repair [66–69]. Important dose-, time-, and tissue-specific differences in the effects of As, as well as important gene-environment and coexposure interactions, have been reported in several studies [70–75]. The effect of As on lipid and amino acid metabolism has been previously demonstrated [41], intimately associated with fatty acid beta-oxidation and amino acid metabolic abnormalities.

In our study, we focused mainly on the lipid alterations after chronic and intergenerational exposure to low doses of arsenic in drinking water. In this lipidomic study, we identified principally three classes of lipids that were significantly altered: glycerophospholipids (lysophospholipids and phosphatidylcholines), glycerolipids (triglycerides), and sterol lipids (3-deoxyvitamin D_3_).

The effect of As on lipid metabolism has been demonstrated in different models. Carlson and colleagues demonstrated, in zebrafish exposed to As, that two genes involved in lipid transport/metabolism (carnitine O-octanoyltransferase (*crot*) and 3-hydroxy-3-methylglutaryl-CoA synthase 1 (*hmgcs1*)) were responsive after 7 days of exposure to 10 ppb sodium arsenite in water [76]. Sex-specific and duration-dependent responses were also observed [77]. In addition, Garciafigueroa demonstrated that 5-week exposure of mice to 100 *μ*g/L of arsenic in drinking water reduced the adipose tissue expression of perilipin (PLIN1, a lipid droplet coat protein), which regulates lipid storage and lipolysis [20].

In relation to the glycerophospholipids, we observed a decrease in the levels of different phosphatidylcholines (PC) in a sex-specific manner, that is, only in AsF, and an increase in the level of lysophosphatidylcholines (LysoPCs). LysoPCs are generated by free radical-catalyzed oxidation of polyunsaturated PCs to oxidatively truncated phosphatidylcholines (oxPCs); oxPCs are hydrolysed by the platelet-activating factor acetylhydrolase, a phospholipase (PL) A2 that exists in plasma associated with LDL [78]. Under pathological conditions, overstimulation of phospholipase A results in breakdown of the PC membrane and subsequent accumulation of LysoPCs. LysoPCs can also be formed by the action of lecithin-cholesterol acyltransferase (LCAT) in plasma. Wang and colleagues reported a marked increase in the level of LysoPCs and elevated expression of hepatic *lcat* in male rats treated with 10 ppm arsenic, suggesting that arsenic exposure may disrupt the transformation process of LysoPCs, leading to their accumulation [41].

In general, lysophospholipids are involved in many physiological processes, including inflammation [79]. LysoPCs have a role as proinflammatory molecules, attracting thromboxanes, leukotrienes, and prostaglandins. Elevated levels of LysoPCs have been linked to the cardiovascular complications associated with diabetes [80], atherosclerosis, ischaemia [78], renal failure during hemodialysis [81], rheumatoid arthritis [82], asthma [83], sepsis [84], hyperlipidaemia [85], endometriosis [86], and psoriasis [87].

With regard to the modulation in glycerolipid metabolism, the results are not conclusive. Several studies have suggested the implication of arsenic in metabolic dysregulation, based on the effect of arsenic exposure on triglyceride production and lipid metabolism [88]. In our study, three identified species of glycerolipids (TG) were differentially regulated (one downregulated and two upregulated) between female and male rats exposed to As (Table 1). The level of expression of one of these TG was significantly different between female and male rats.

When we analysed the global metabolic perturbation observed in the present work and its relation to As exposure, an increase was observed in a metabolite putatively predicted to be 3-demethylubiquinol, which is found in the ubiquinol biosynthesis pathway [89]. It is interesting that ubiquinone derivatives, including 3-demethylubiquinol-10 (reduced form of coenzyme Q10) and 6-methoxy-3-methyl-2-all-trans-decaprenyl-1,4-benzoquinol, were upregulated, suggesting that mitochondria were undergoing substantial oxidative stress, which eventually leads to cell death. An increase in 3-demethylubiquinol could suggest inhibition of 3-demethylubiquinol 3-O-methyltransferase by a mechanism of negative feedback, interrupting the synthesis of ubiquinol-10, which is the reduced form of ubiquinone and responsible for preventing lipid oxidation. Recent *in vitro* and *in vivo* studies have provided evidence that coenzyme Q10 is involved in inflammatory processes and lipid metabolism via gene expression changes [90].

In the present work, we also demonstrated that lipid oxidation was increased in the serum of arsenic exposed rats (Figure 8). It has been described that oxidative modifications of lipids occur during inflammatory processes and lead to the formations and accumulations of oxidized lipids that produce cellular reactions such as apoptosis and chronic inflammatory reactions, which are considered critical factors for most chronic diseases. Oxidized lipids in cellular membrane and lipoproteins have been considered as an endogenous class of damage-associated molecular patterns (DAMPs), which share common structural motifs with microbial pathogen-associated molecular patterns (PAMPs). Thus, oxidized lipids may activate the same pattern recognition receptors (PRRs) on immune and vascular cells. It has been increasingly recognized that elicitation of innate immune response by these oxidized lipids can be physiological (homeostatic) or pathophysiological (adverse) depending on the biological context and duration of the immune activation. In this work, we do not have any immunological marker, but it will be considered in future investigations [91]. Lipid oxidation-derived products are key players in the initiation and progression of atherosclerotic lesions and inflammatory process. Oxidized lipids, derived from oxidatively modified low-density lipoproteins (LDLs), which accumulate in the intima, strongly modulate inflammation-related gene expression, through involvement of various signalling pathways [92].

Arsenic increases lipid oxidation in the liver, kidney, and heart associated with a depletion of GSH [93]. We could speculate that a chronic inflammatory process resulting from As exposure is responsible for the metabolic changes found in lipid metabolism.

Glycolipids, phospholipids (PLs), and cholesterol (Ch) are targets of potential lethal oxidative modification. Under toxic conditions, such as exposure, the extent of oxidative damage overwhelms cellular repair capacity, and the cells induce apoptosis or necrosis, facilitating the development of various pathological states and accelerated ageing. Phosphatidylcholine hydroperoxide (PCOOH) is the primary product of lipid oxidation: it undergoes nonenzymatic reactions, leading to the formation of 4-hydroxynonenal (4-HNE) and malondialdehyde (MDA), secondary products of lipid oxidation [94]. These lipid oxidation products induce oxidative stress and are involved in the pathogenesis of a number of degenerative diseases; consequently, they are considered to be biomarkers of oxidative stress. LysoPCs are one of the numerous lipid products formed during the oxidation of low-density lipoprotein (LDL).

Another important finding in our study is the predictive dysregulation of thyroid hormone metabolism. As is a potent endocrine disruptor, altering steroid hormone receptor- (SR-) mediated gene regulation for all five steroid receptors (SRs) (i.e., the receptors for glucocorticoid (GR), androgen (AR), progesterone (PR), mineralocorticoid (MR), and oestrogen (ER)) and two members of the larger nuclear hormone receptor superfamily, the retinoic acid (RA) receptor (RAR) and the thyroid hormone (TH) receptor (TR), at very low concentrations in cell culture and in whole-animal models [72, 73, 95, 96]. Thyroid hormones (THs) are also a major factor controlling metabolic rates in mammals; these hormones stimulate both lipogenesis and lipolysis, thereby provoking thermogenesis and an increase of the rate of both anabolic and catabolic reactions dependent on time and substrate. On the contrary, fat metabolism is affected by THs. The increase in TH levels modifies the composition of membrane phospholipids [97], increasing the degree of unsaturation particularly in the mitochondrial membranes [88], making them more susceptible to free radical attack [98] and resulting in augmented lipid oxidation in mitochondria.

Important differences in the levels of expression of metabolites were found in our work between AsM and AsF rats. Considering the effect of arsenic as an endocrine disruptor [95], these differences could be attributed to oestrogen hormones, although this could not be demonstrated here. Different histone methylation patterns have been observed between men and women after arsenic exposure [99]. Additionally, a recent *in vivo* study demonstrated sex-specific metabolomic changes in As (+3 oxidation state) methyltransferase (As3mt)-knockout mice exposed to iAs, raising the possibility of a role for sex hormones in the regulation of arsenite-methyltransferase-catalyzed arsenic metabolism [38].

In the study of biochemical alterations related to arsenic exposure, animal models are a powerful tool. However, there is no ideal model for reproducing human real exposure and the effects caused on metabolic perturbations. For this reason, it is necessary to mention some of the straights and limitations of the animal model employed in the present work. Rats do have metabolic, distribution, and excretion functions similar to those in humans and have been valuable models for investigating the effects of arsenic. However, extrapolating the rat model to humans requires an understanding of the differences between the metabolism and toxicokinetics of arsenicals in these species. Rats have longer retention time of arsenic in the blood because arsenic binds to rat hemoglobin (due to an extra cysteine on the a-globin chain of the hemoglobin, which has a strong affinity to trivalent arsenic) [100]. In another study, it was demonstrated that hepatocytes from rats, macaques, and dogs had much greater capacities to methylate iAs than do hepatocytes from humans, mice, or rabbits [101].

## 6. Conclusions

The results obtained in our work demonstrate a clear effect of As exposure on lipid metabolism and related pathways in a model of chronic and intergenerational exposure. The prolonged As exposure, before and during pregnancy and during the neonatal development, could result in lipid oxidation and the degradation of membrane lipids, which was more evident in male rats in comparison to female rats. Thus, serum phospholipids might be biomarkers for exposure to As; however, these results need to be extrapolate in future studies conducted in humans.

## Figures and Tables

**Table 1:** Identified differential metabolites in rat serum.

Component name	Observed *m/z*	Mass error (ppm)	Retention time (min)	Adducts	MS^E^ fragments	Fold change (*p* value)^a^	
						AsF vs CF	AsM vs CM
LysoPC (20:4)	544.3395	−0.5	0.99	+H	184.07268, 258.10896	+3.71 (0.32231)	+1.4 (0.021)
LysoPC (15:0)	482.3238	−0.7	1.05	+H	184.10804, 258.10896	+1.1 (0.69)	+2.44 (6.05*E* − 06)^b,c^
LysoPC (16:0)	496.3397	0.0	1.22	+H	184.07273, 258.1101	+15 (0.225)	+3.38 (7.27*E* − 05)^b^
LysoPC (18:1)	522.3552	−0.5	1.27	+H	184.07273, 258.1101	+191 (0.098)	+1.77 (1.4*E* − 04)^b^
PC (O-16:0/1:0)	510.3555	0.2	1.43	+H	492.34431	−1.0 (0.988)	+2.54 (4.94*E* − 06)^b,c^
PC (20:4/16:0)	782.5709	5.0	6.21	+Na	184.07263, 496.33883	−10000 (5.27*E* − 03)	−1.5 (0.034)
PC (16:0/18:2)	758.5711	2.2	6.51	+H	184.07266, 496.33862, 502.32807	−2.03 (0.09)	−1.8 (0.043)
TG (16:0/18:1/20:1)	904.8347	2.0	16.03	+NH4	631.56710, 605.55007, 577.51907	−1.4 (0.08)	−1.6 (2.34*E* − 03)
TG (16:0/18:3/22:5)	920.7724	2.5	14.96	+NH4	647.5034, 625.5191, 573.48693	+1.5 (0.032)	+1.3 (0.06)
TG (16:1/18:2/22:6)	918.7576	−9.5	14.57	+H	647.49851, 621.48725, 573.48690	+1.7 (9.52*E* − 03)	+1.0 (0.97)
3-Deoxyvitamin D3	369.3510	−0.1	15.72	+H	287.23, 233.2252, 215.1783, 161.1315, 147.1159	+1.5 (0.0624)	+1.61 (4.0*E* − 04)^b^

^a^FC, fold change in the specified comparisons. “+” indicates upregulation and “−” indicates downregulation. ^b^*p* values significant also due to FDR. ^c^*p* values significant also to Bonferroni test correction.

## Data Availability

The metabolomic raw data used to support the findings of this study are available from the corresponding author upon request.

## Supplementary Materials

Supplementary Materials Supplementary Figure 1: base peak UPLC-TOF MS chromatogram of a rat serum extract using electrospray ionization in positive mode; different lipid classes elute in different elution time windows. For the experimental details, see Materials and Methods. PC: phosphatidylcholines; PG: phosphatidylglycerol; PE: phosphatidylethanolamine; SM: sphingomyelin; DG: diacylglycerides; CE: cholesterol esters; TG: triacylglycerides. Supplementary Figure 2: overlaid chromatograms of quality controls (QC) showing good reproducibility for retention time, peak shape, and peak intensity. (a) QCFCT: overlaid chromatograms of three replicates of QC from female control group. (b) QCFAS: overlaid chromatograms of three replicates of QC from female exposed group. (c) QCMCT: overlaid chromatograms of three replicates of QC from male control group. (d) QCMAS: overlaid chromatograms of three replicates of QC from female control group. The conditions employed for QC acquisition data were the same for the samples. Supplementary Figure 3: heat map visualization constructed based on the differential metabolites of importance for the sera of As-treated groups. The heat map was constructed based on the potential candidates of importance, which were extracted with PLS-DA analysis. Rows, metabolites; columns, samples. Colour key indicates metabolite expression value: lowest (green) and highest (red). Variable differences are revealed between the control groups and As-exposed and between sexes.
