# Supplementary figures and images for: Lipid Metabolism Alterations in a Rat Model of Chronic and Intergenerational Exposure to Arsenic
Source: Biomed Res Int. 2019 Oct 15;2019:4978018. doi: 10.1155/2019/4978018 (PMC6815581; doi:10.1155/2019/4978018)

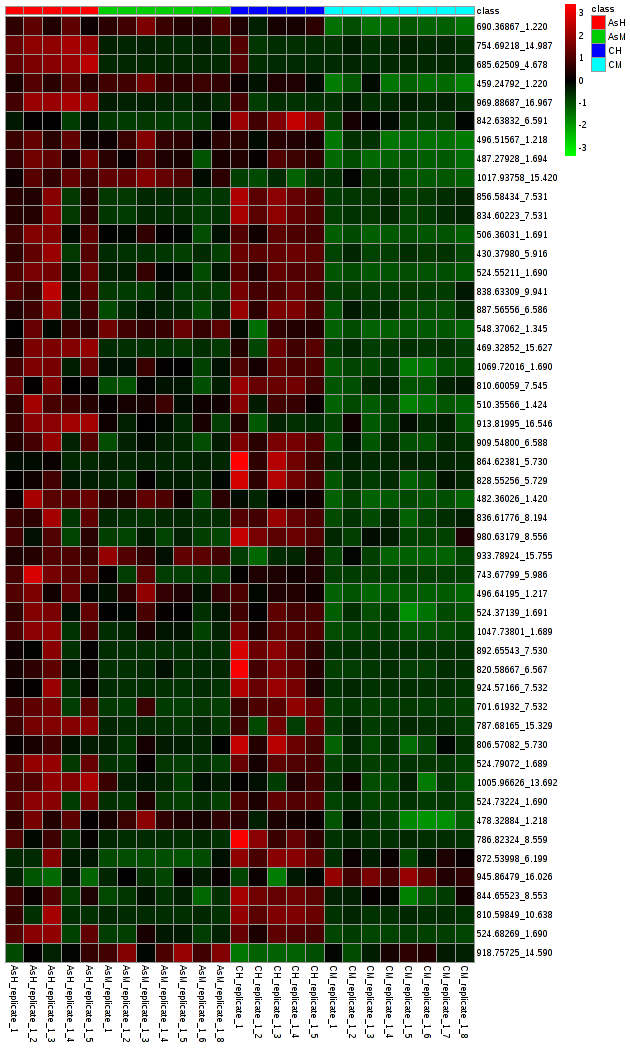

Supplement: Supplementary Materials — Supplementary Figure 1: base peak UPLC-TOF MS chromatogram of a rat serum extract using electrospray ionization in positive mode; different lipid classes elute in different elution time windows. For the experimental details, see Materials and Methods. PC: phosphatidylcholines; PG: phosphatidylglycerol; PE: phosphatidylethanolamine; SM: sphingomyelin; DG: diacylglycerides; CE: cholesterol esters; TG: triacylglycerides. Supplementary Figure 2: overlaid chromatograms of quality controls (QC) showing good reproducibility for retention time, peak shape, and peak intensity. (a) QCFCT: overlaid chromatograms of three replicates of QC from female control group. (b) QCFAS: overlaid chromatograms of three replicates of QC from female exposed group. (c) QCMCT: overlaid chromatograms of three replicates of QC from male control group. (d) QCMAS: overlaid chromatograms of three replicates of QC from female control group. The conditions employed for QC acquisition data were the same for the samples. Supplementary Figure 3: heat map visualization constructed based on the differential metabolites of importance for the sera of As-treated groups. The heat map was constructed based on the potential candidates of importance, which were extracted with PLS-DA analysis. Rows, metabolites; columns, samples. Colour key indicates metabolite expression value: lowest (green) and highest (red). Variable differences are revealed between the control groups and As-exposed and between sexes. [file 4978018.f1.zip › 4978018.f1/FIG A3.png]
